# Supplementary material for: FTD-tau S320F mutation stabilizes local structure and allosterically promotes amyloid motif-dependent aggregation
Source: Nat Commun. 2023 Mar 23;14:1625. doi: 10.1038/s41467-023-37274-6 (PMC10036635; doi:10.1038/s41467-023-37274-6)
Supplement: Supplementary file 1 — Supplementary Information [file 41467_2023_37274_MOESM1_ESM.pdf]

SUPPLEMENTARY INFORMATION

FTD-tau S320F mutation in tau stabilizes local structure and allosterically promotes amyloid motif-dependent aggregation

Dailu Chen[1,2,5], Sofia Bali[1,2,5], Ruhar Singh[2], Aleksandra Wosztyl[2], Vishruth Mullapudi[2], Jaime Vaquer-Alicea[2], Parvathy Jayan[2], Shamiram Melhem[3], Harro Seelaar[3], John C. van Swieten[3], Marc I. Diamond[2], Lukasz A. Joachimiak[2,4,#]

SUPPLEMENTARY TABLES

Collected parameter	Voltage	Excitation (nm)	Emission filter (nm)
Forward Scatter	340	488	-
Side Scatter	190	488	488/10 BP
Acceptor, "mCherry"	340	561	595 LP<br>610/20 BP
Donor, "Alexa Fluor 488"	200	488	505 LP<br>530/30 BP
FRET, "PerCP"	340	488	595 LP<br>610/20 BP

Supplementary Table 1. Parameters used on flow cytometry data collection of tauRD-mEOS3.2 expression system.

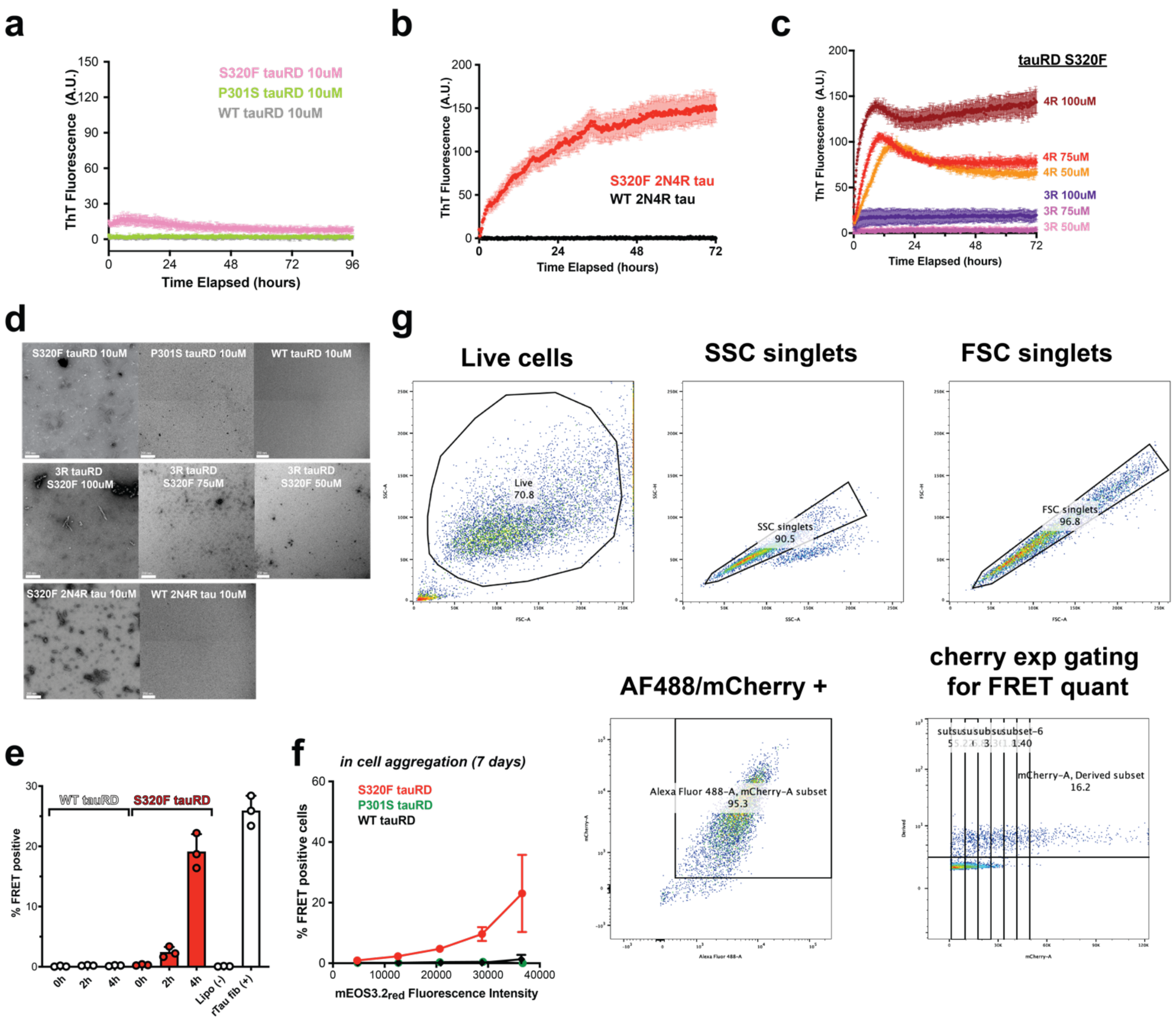

Supplementary Figure 1. ***In vitro*** **and in cell assays on tauRD (4R), 2N4R tau, and tauRD (3R).** **a.** ThT fluorescence assay on S320F (red and magenta), P301S (green and olive), and WT (black and gray) tauRD at 62.5μM and 25μM, 37 °C. **b.** ThT fluorescence assay of full-length 2N4R tau S320F (red) and WT (black). **c.** ThT fluorescence assay on tauRD (4R) and tauRD (3R) S320F. Light to dark colors indicate increasing concentrations. Aggregation experiments were performed in triplicate, and the averages are shown with standard deviation. The data were fitted to a non-linear regression model in GraphPad Prism to estimate an average $t_{1/2max}$ with a standard deviation. **d.** TEM images of the endpoint ThT assay on the conditions mentioned above. The black bars represent 200 nm distance. **e.** WT and S320F tauRD incubated at 2 hr, 4 hr, or without incubation were transduced in parallel with lipofectamine (negative control) and tau fibrils (positive control) into the tau biosensor cells. FRET signal indicating the amount of tau aggregates from each condition (tau RD-CFP/tau RD-YFP) was measured by flow cytometry on three biological triplicates of at least 10,000 cells per condition. Data is shown as an average across three experiments, with error bars representing a 95% CI of each condition. **f.** HEK293T cells expressing S320F, P301S, or WT tauRD-mEOS3.2 were fixed on Day 7.

FRET (tauRD-CFP/tauRD-mCherry) was measured by flow cytometry on three biological triplicates of at least 10,000 cells per condition on S320F (red), P301S (green), or WT (black) at 7 days. Comparison was conducted at multiple fluorescent intensity levels of mEOS3.2_{red}. Data is shown as an average across three experiments, with error bars representing a 95% CI of each condition. **g.** Gating strategy to extract live, single, Alexa-Fluor 488 and mCherry double-positive cells and expression level for FRET quantification.

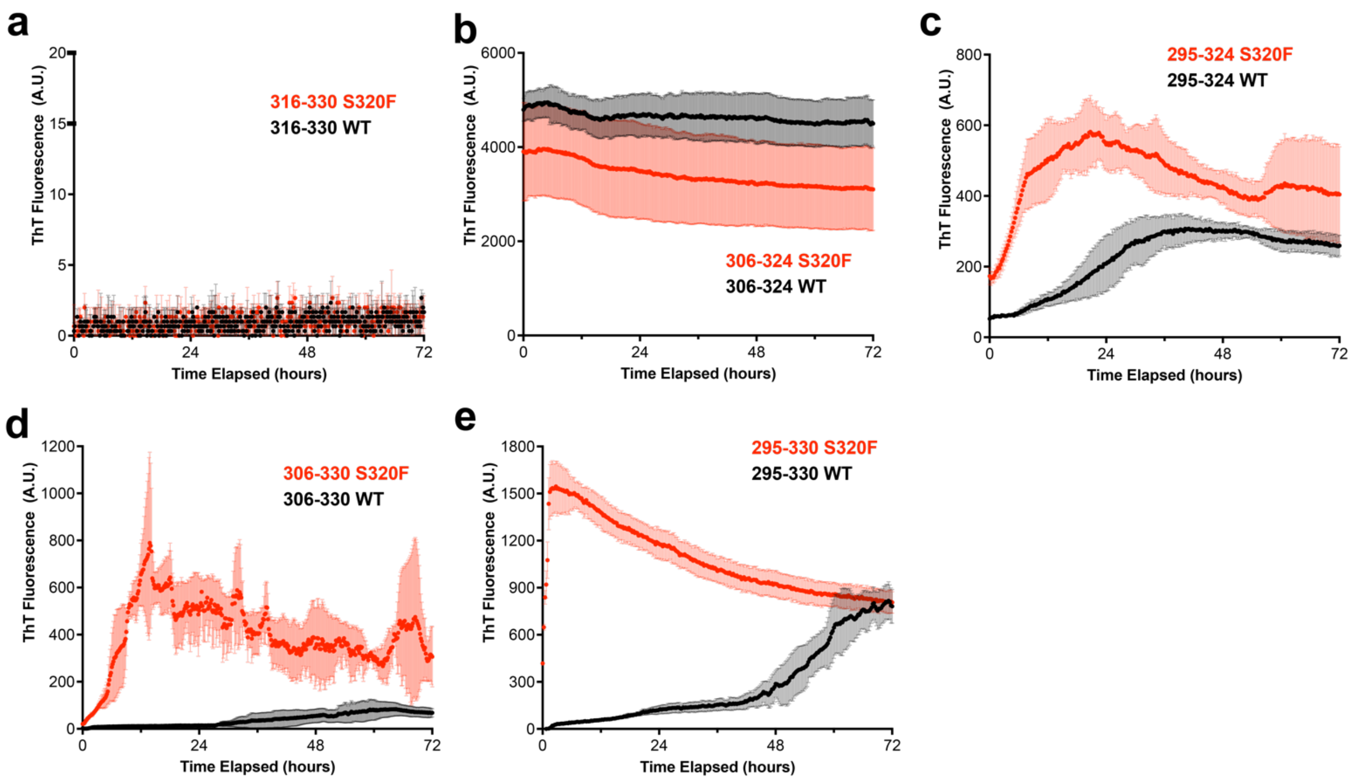

Supplementary Figure 2. VQIVYK amyloid motif is essential in S320F facilitated aggregation. Raw ThT curves comparing WT and S320F fragments: (**a**) 316-330, b 306-324, (**c**) 295-324, (**d**) 306-330, and (**e**) 295-330. Aggregation experiments were performed in triplicate, and the averages are shown with standard deviation. The data were fitted to a non-linear regression model in GraphPad Prism to estimate an average $t_{1/2max}$ with a standard deviation.

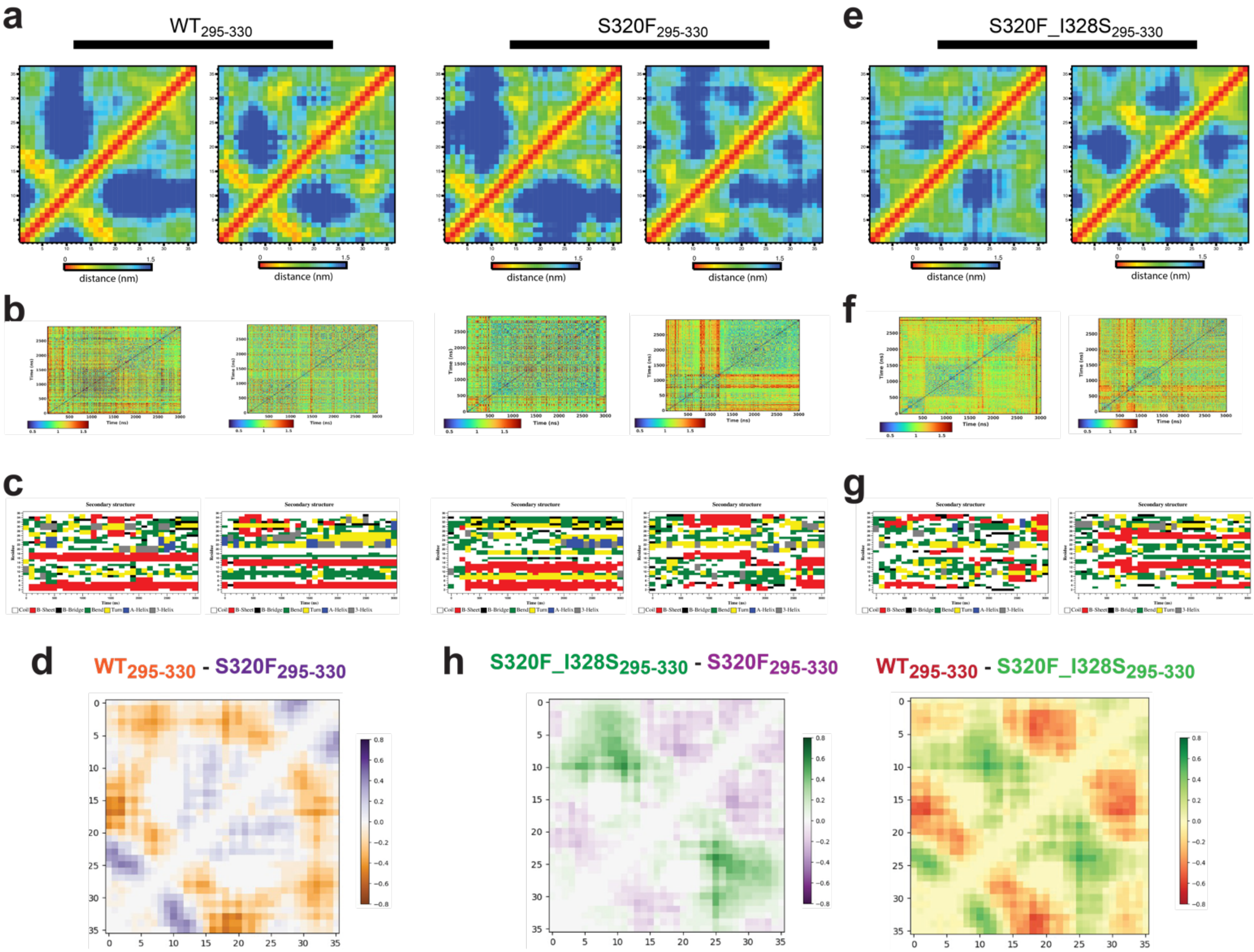

Supplementary Figure 3. Validation of replicate MD trajectories. a. Contact maps for WT$_{295\text{-}330}$ (left) and S320F$_{295\text{-}330}$ (right) simulations for two 3 μs replicate trajectories with cutoff (0.6 nm) applied to the center for mass of side chains. The color bar of the contact map indicates the distance between pairs of residues in the range of 0 – 1.5 nm. **b.** Two-dimensional RMSDs comparison of structures in the MD trajectory calculated between all pairs of conformations visited within replicate WT$_{295\text{-}330}$ (left) and S320F$_{295\text{-}330}$ (right) MD simulations. The color bar of the plot indicates the distance between two conformations in the range of 0 – 1.5 nm. **c.** Time-dependent secondary structure analysis using DSSP for two replicate 3 μs trajectories for WT$_{295\text{-}330}$ (left) and S320F$_{295\text{-}330}$ (right). Coil, beta-sheet, beta-bridge, bend, turn, alpha-helix, and 3-helix are colored white, red, black, green, yellow, blue, and grey, respectively. **d.** Difference contact map between cumulative 15us WT$_{295\text{-}330}$ and S320F$_{295\text{-}330}$ trajectories. Orange and purple regions indicate regions stronger in the WT and S320F ensemble, respectively. **e-g.** Contact plots, 2D RMSD plots, and DSSP analysis for two replicate trajectories for S320F_I328S$_{295\text{-}330}$ (same as a-c above). **h**. Difference contact maps calculated for S320F_I328S$_{295\text{-}330}$ and S320F$_{295\text{-}330}$ (left) and WT$_{295\text{-}330}$ and S320F_I328S$_{295\text{-}330}$ (right). Green colors indicate regions that are closer in the S320F_I328S$_{295\text{-}330}$ ensemble, while purple and red indicate regions that are closer in the S320F (left) and WT (right), respectively.

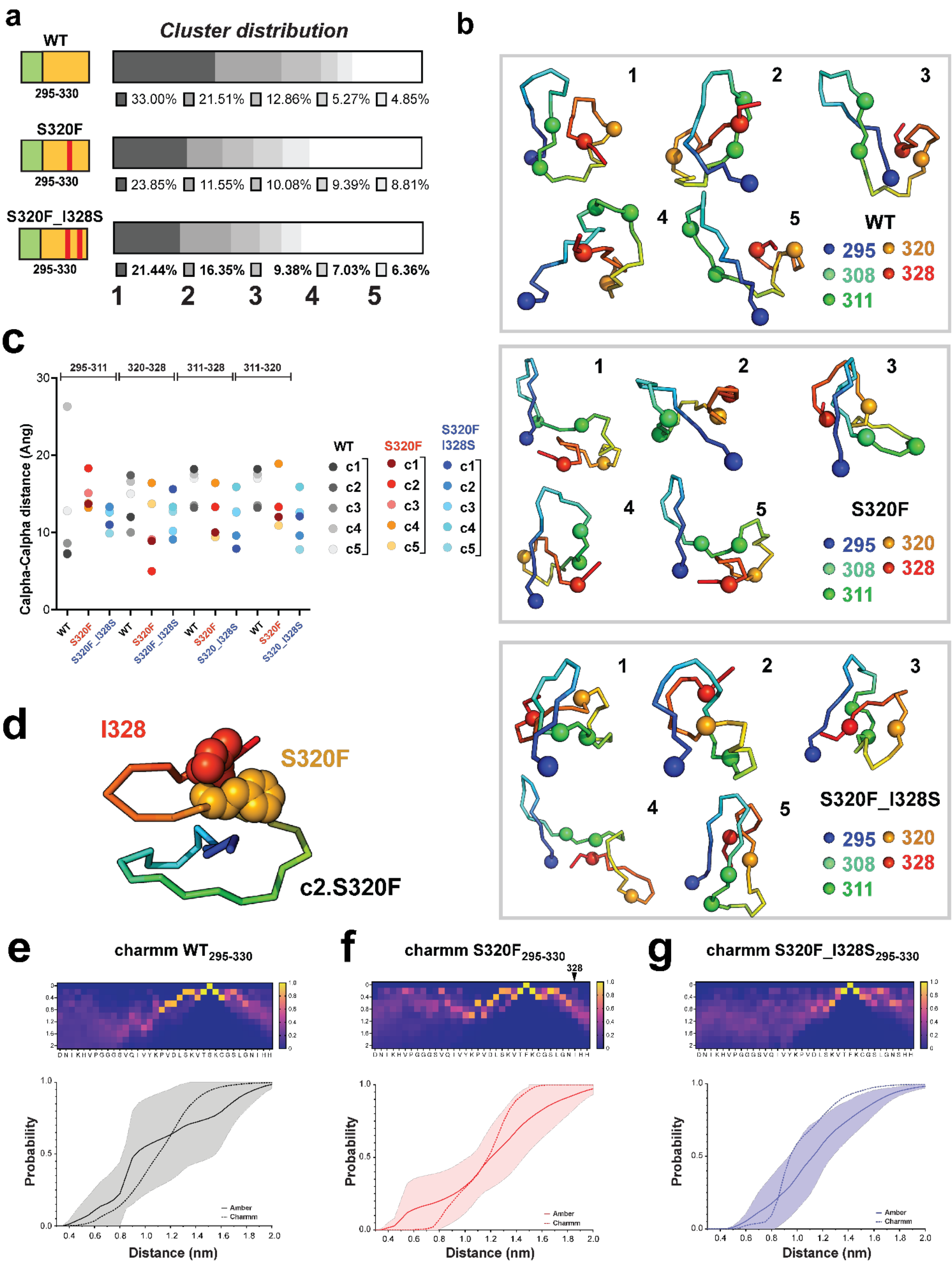
a
Cluster distribution
WT
295-330
33.00% 21.51% 12.86% 5.27% 4.85%
S320F
295-330
23.85% 11.55% 10.08% 9.39% 8.81%
S320F_I328S
295-330
21.44% 16.35% 9.38% 7.03% 6.36%
1 2 3 4 5
b
WT
S320F
S320F_I328S
295 320
308 328
311
c
295-311 320-328 311-328 311-320
Calpha-Calpha distance (Ang)
WT c1 c2 c3 c4 c5
S320F c1 c2 c3 c4 c5
S320F I328S c1 c2 c3 c4 c5
d
I328
S320F
c2.S320F
e
charmm WT$_{295-330}$
f
charmm S320F$_{295-330}$
328
g
charmm S320F_I328S$_{295-330}$
Probability
Distance (nm)
Amber
Charmm

Supplementary Figure 4. MD simulations on $WT_{295\text{-}330}$, $S320F_{295\text{-}330}$, and S320F_$I328S_{295\text{-}330}$ reveal rearrangements in nonpolar clustering. a. Cluster distribution of MD simulations on $WT_{295\text{-}330}$, $S320F_{295\text{-}330}$, and S320F_$I328S_{295\text{-}330}$ using a 0.6 nm RMSD cutoff. The percentages for the top five clusters are shown. **b.** The mean structure of each of the top five clusters for WT, S320F, and S320F_I328S. C-alpha atoms for 295, 308, 311, 320 and 328 are shown as spheres and colored blue, cyan, green, orange and red, respectively. Structures are shown in ribbon and colored blue (N-term) to red (C-term). **c.** Cα distance measured of the pairs of interest for the mean structures of the top five clusters. c1 = cluster 1, etc. WT is colored from black to gray, S320F is colored red to pink, and S320F_I328S is colored from blue to cyan from cluster 1 to cluster 5. **d**. Model representing cluster 2 from the $S320F_{295\text{-}330}$ simulation (i.e., c2.S320F) illustrating stabilizing interactions between F320-I328. F320 and I328 are shown as spheres, and the rest of the peptide is shown as ribbon and colored from blue (N-term) to red (C-term). (Top) Cα distance distribution of each residue to the S320 position in WT(**e**), S320F (**f**), and S320F_I328S (**g**) single 3us trajectory using the CHARMM force field. The color scheme indicates the % population (scale 0-1) of the residue at a particular distance to 320. Arrows point to the positions/regions of interest. (Bottom) Cumulative distance distributions comparing the average (solid line) +/- SD (shaded area) distances between the center of mass of residue 320 and center of mass of residue 328 for 5 replicates from 3us in AMBER from WT, S320F, and S320F_I328S compared to the equivalent distributions for one 3us replicate from CHARMM (dashed line).

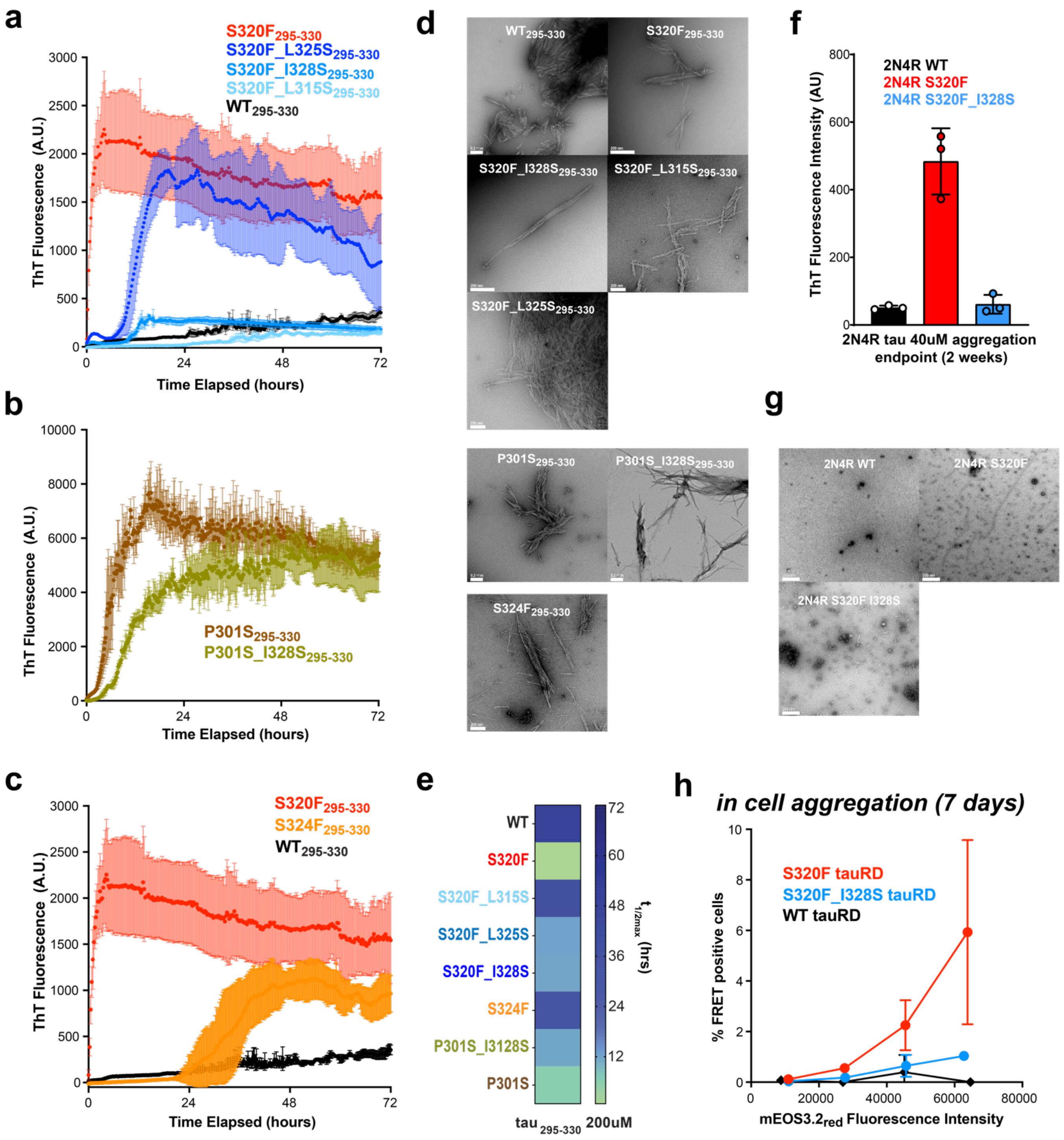

Supplementary Figure 5. Aggregation assays of mutant peptide controls and cell model assay on Day 7. a. ThT fluorescence assay on S320F$_{295\text{-}330}$ (red), S320F_L325S$_{295\text{-}330}$ (dark blue), S320F_I328S$_{295\text{-}330}$ (blue), S320F_L315S$_{295\text{-}330}$, and WT$_{295\text{-}330}$ (black) at 200μM, 37 °C. **b.** ThT fluorescence assay on P301S$_{295\text{-}330}$ (brown) and P301S_I328S$_{295\text{-}330}$ (olive) at 200 μM, 37 °C. **c.** ThT fluorescence assay on S320F$_{295\text{-}330}$ (red), S324F$_{295\text{-}330}$ (orange), and WT$_{295\text{-}330}$ (black) at 200μM, 37 °C. The S324F condition was tested in the same experiment as in **a,** and thus S320F and WT are used again here. The data are presented as an average $t_{1/2}$max +/-SD from fits to a non-linear regression

model in GraphPad3 Prism n=3 biological replicates. **d.** Representative TEM images of the endpoint ThT assay on 295-330 sequence fragments from **(a-c)**. **e**. Summary of $t_{1/2max}$ values calculated from triplicate ThT aggregation curves for WT, S320F, S320F_L315S, S320F_L325, S320F_I328S, S324F, P301S_I328S, and P301S tau295-330 peptides. $T_{1/2max}$ values are colored from teal to blue. The constructs are labeled as in (**a-c**). **f.** Aggregation assay endpoint ThT fluorescence intensity of 40μM FL 2N4R WT (black), 2N4R S320F (red), 2N4R S320F_I328S (blue). Data are shown as the average +/- SD of the endpoint after 2 weeks of aggregation for n=3 biological replicates. **g.** Representative TEM images of endpoint aggregation assay on FL 2N4R WT, S320F, and S320F_I328S tau constructs at 40μM **(f)**. **h.** HEK293T cells expressing S320F, S320F_I328S, or WT tauRD-mEOS3.2 were fixed on Day 4. FRET (tauRD-CFP/tauRD-mCherry) was measured by flow cytometry on three biological triplicates of at least 10,000 cells per condition on S320F (red), P301S (green), or WT (black) at 7 days. Comparison was conducted at multiple fluorescent intensity levels of $mEOS3.2_{red}$. Data is shown as an average across three experiments, with error bars representing a 95% CI of each condition.

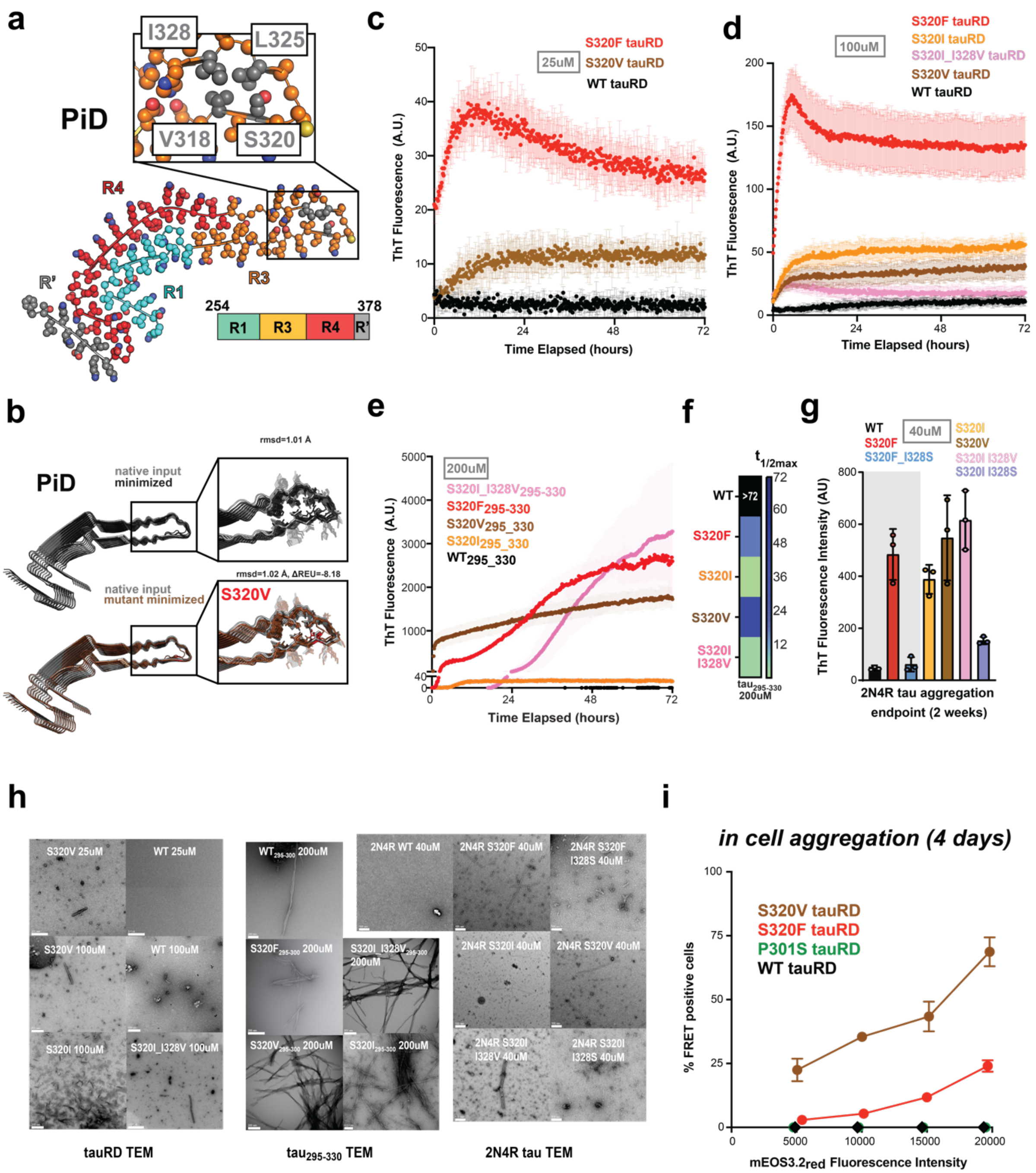

Supplementary Figure 6. Computationally designed tau sequences derived from the PiD fibril scaffold aggregate spontaneously *in vitro* and in cells. a. PiD cryo-EM tau fibril (atomic model, top-view). Structure is shown in sphere representation and is colored by repeat domain as in Fig 1. In-set view of residues in proximity to S320 (L325 and I328) shown as spheres and colored in gray. **b.** Energy-minimized models of S320V 9-mer (brown), WT 9-mer overlaid to WT native input in PiD tau fibril structure. **c.** ThT fluorescence assay on S320F (red), S320V (brown), and WT (black) tauRD at 25µM, 37 °C. WT and S320F tauRD data is reused from Fig. 5e. **d.** ThT fluorescence assay on S320F (red), S320I (orange), S320I_I328V (pink), S320V (brown), and WT (black) tauRD at 100µM, 37 °C. **e.** ThT fluorescence assay on S320F$_{295\text{-}330}$ (red), S320I$_{295\text{-}330}$ (orange), S320I_I328V$_{295\text{-}330}$ (pink), and S320V$_{295\text{-}330}$ (brown) at 200µM, 37 °C. The data are presented as an average with SD from biological

triplicates. **f.** Summary of average $t_{1/2max}$ values (teal to blue) calculated from (**e**) using linear-regression model in GraphPad3 Prism. **g**. Endpoint ThT fluorescence intensity from aggregation of 40uM FL 2N4R WT (black), 2N4R S320F (red), 2N4R S320F_I328S (blue) S320I (orange), S320V (brown), S320I_I328V (pink), and S320I_I328S (purple). FL 2N4R WT, S320F, and S320F_I328S tau aggregation data are reused from Supplementary Fig. 5f. Data are shown as the average +/-SD for biological triplicates. **h**. Representative TEM images of aggregation reactions from (**c**-**e** and **g**). **i**. HEK293T cells expressing S320V, S320F, P301S, or WT tauRD-mEOS3.2 were fixed on Day 4. FRET (tauRD-CFP/tauRD-mCherry) measured by flow cytometry >10,000 cells per condition on S320V (brown), S320F (red), P301S (green), or WT (black). Comparison was conducted at multiple fluorescent intensity levels of $mEOS3.2_{red}$. Data are shown as averages with 95% CI across biological triplicates.
