# Supplementary material for: FTD-tau S320F mutation stabilizes local structure and allosterically promotes amyloid motif-dependent aggregation
Source: Nat Commun. 2023 Mar 23;14:1625. doi: 10.1038/s41467-023-37274-6 (PMC10036635; doi:10.1038/s41467-023-37274-6)
Supplement: Supplementary file 3 — Reporting Summary [file 41467_2023_37274_MOESM3_ESM.pdf]

nature portfolio

Corresponding author(s): Joachimiak LA

Last updated by author(s): 2023-02-28

Reporting Summary

Nature Portfolio wishes to improve the reproducibility of the work that we publish. This form provides structure for consistency and transparency in reporting. For further information on Nature Portfolio policies, see our Editorial Policies and the Editorial Policy Checklist.

Please do not complete any field with "not applicable" or n/a. Refer to the help text for what text to use if an item is not relevant to your study.
For final submission: please carefully check your responses for accuracy; you will not be able to make changes later.

Statistics

For all statistical analyses, confirm that the following items are present in the figure legend, table legend, main text, or Methods section.

n/a	Confirmed	
☐	☒	The exact sample size (*n*) for each experimental group/condition, given as a discrete number and unit of measurement
☐	☒	A statement on whether measurements were taken from distinct samples or whether the same sample was measured repeatedly
☒	☐	The statistical test(s) used AND whether they are one- or two-sided<br>*Only common tests should be described solely by name; describe more complex techniques in the Methods section.*
☒	☐	A description of all covariates tested
☒	☐	A description of any assumptions or corrections, such as tests of normality and adjustment for multiple comparisons
☐	☒	A full description of the statistical parameters including central tendency (e.g. means) or other basic estimates (e.g. regression coefficient) AND variation (e.g. standard deviation) or associated estimates of uncertainty (e.g. confidence intervals)
☒	☐	For null hypothesis testing, the test statistic (e.g. F, t, r) with confidence intervals, effect sizes, degrees of freedom and P value noted<br>*Give P values as exact values whenever suitable.*
☒	☐	For Bayesian analysis, information on the choice of priors and Markov chain Monte Carlo settings
☒	☐	For hierarchical and complex designs, identification of the appropriate level for tests and full reporting of outcomes
☒	☐	Estimates of effect sizes (e.g. Cohen's *d*, Pearson's *r*), indicating how they were calculated

Our web collection on statistics for biologists contains articles on many of the points above.

Software and code

Policy information about availability of computer code

Data collection	ThT data were acquired on a Tecan Spark platereader (https://lifesciences.tecan.com/multimode-plate-reader). FRET analysis on aggregation of tauRD mutants in cells was acquired on a BD Fortessa flow cytometer. TEM images were acquired on a FEI Tecnai G2 Spirit Biotwin microscope. MD simulations were performed using GROMACS-5.04 (available http://www.gromacs.org). Tau fibril dREU design calculations were carried out with ROSETTA v3.12 (available at https://www.rosettacommons.org/).
Data analysis	All images of structures were produced in pymol v1.8.4.2. All plots were generated with GraphPad Prism 9.4.1. FRET data was analyzed using FlowJo v10 (available at https://www.flowjo.com/solutions/flowjo/downloads).

For manuscripts utilizing custom algorithms or software that are central to the research but not yet described in published literature, software must be made available to editors and reviewers. We strongly encourage code deposition in a community repository (e.g. GitHub). See the Nature Portfolio guidelines for submitting code & software for further information.

Data

Policy information about availability of data

All manuscripts must include a data availability statement. This statement should provide the following information, where applicable:

- Accession codes, unique identifiers, or web links for publicly available datasets
- A description of any restrictions on data availability
- For clinical datasets or third party data, please ensure that the statement adheres to our policy

All ThT, cell-based aggregation, MD, and XLMS data are available as source data 1, source data 2, source data 3, and source data 4, respectively. Source data is also available on zenodo under accession number 7668320 [https://doi.org/10.5281/zenodo.7668320]. Raw MS data used for the XL-MS analysis has been deposited in the MassIVE and ProteomeXchange databases under the accession numbers MSV000091047 [https://massive.ucsd.edu/ProteoSAFe/dataset.jsp?accession=MSV000091047] and PXD040126 [http://proteomecentral.proteomexchange.org/cgi/GetDataset?ID=PXD040126], respectively. Raw MD trajectories, mass-spectrometry and Rosetta models are available as supplementary data and have been deposited in zenodo under accession number 7668320 [https://doi.org/10.5281/zenodo.7668320]. PDB ids used in the study include 6tjo [http://doi.org/10.2210/pdb6tjo/pdb] and 6gx5 [http://doi.org/10.2210/pdb6GX5/pdb].

Human research participants

Policy information about studies involving human research participants and Sex and Gender in Research.

Reporting on sex and gender	NA
Population characteristics	NA
Recruitment	NA
Ethics oversight	NA

Note that full information on the approval of the study protocol must also be provided in the manuscript.

Field-specific reporting

Please select the one below that is the best fit for your research. If you are not sure, read the appropriate sections before making your selection.

- [x] Life sciences
- [ ] Behavioural & social sciences
- [ ] Ecological, evolutionary & environmental sciences

Life sciences study design

All studies must disclose on these points even when the disclosure is negative.

Sample size	We did not study populations either of animals or humans, thus population sample size is not applicable. In the case of Rosetta dREU calculations we ran 35 independent replicates for each designed mutant (and wild-type) minimization which we and others have shown converges on a solution (Barlow et al. 2018). Using the biohpc computer cluster at UTSW this represented 2 weeks of computer time.
Data exclusions	No data were excluded in the analyses

Replication	Figure 1 and Supplementary Figure 1. The ThT fluorescence aggregation assay was performed as technical triplicates and the data were plotted as averages with standard deviation. The data were fit to a non-linear regression model fitting in GraphPad Prism to estimate an average t1/2max with a standard deviation. TEM images were collected twice. The tau aggregation experiments in cells were carried out as biological triplicates and the FRET levels were reported as averages with standard deviation.<br><br>Figure 2 and Supplementary Figure 2. The ThT aggregation assay was performed as technical triplicates and the data were fit to a non-linear regression model fitting in GraphPad Prism to estimate a t1/2max with a standard deviation. The t1/2max is plotted as averages with standard deviation. TEM images were collected twice.<br><br>Figure 3 and Supplementary Figures 3 and 4. Molecular Dynamic simulations were performed as 5 independent replicate trajectories. The merged trajectories are shown as contact maps. Similarly, from the merged ensembles we calculated distance distributions to residue at position 320. The ensembles were clustered based on similarity and representative structures were selected for the two top 5 clusters. Distances between different residues were used to classify the ensemble below a distance threshold. Representative data from two replicates are shown as contact plots, pairwse rmsd trajectory comparisons and dssp secondary structure analysis. Matching trajectories were performed using the charmm forcefield (3us) and similar distributions were observed of residue contacts in proximity and the cumulative distance distributions for a single charmm replicate were found to be within the deviation observed across the amber replicate data.<br><br>Figure 4 and Supplementary Figure 5. The ThT fluorescence aggregation assay on WT and mutant tauRD, tau peptides and FL 2N4R tau was performed as technical triplicates and the data were plotted as averages with standard deviation. The data were fit to a non-linear regression model fitting in GraphPad Prism to estimate an average t1/2max with a standard deviation. TEM images were collected twice. The tau aggregation experiments in cells were carried out as biological triplicates and the FRET levels were reported as averages with standard deviation. The crosslinking mass spectrometry experiment were performed in 5 technical replicates. The frequency of each high-scored crosslink identified was normalized to the total number of high-scored crosslinks and the average % with standard deviations were plotted in the bar plots. The contact maps show only the average %.<br><br>Figure 5 and Supplementary Figure 6. The Rosetta design calculations at defined positions were substituted for each amino acid using a 9-layer fibirl assembly. Simulations were carried out using 35 replicates to ensure convergence of energies as previously determined (Barlow et al. 2018). Low energy structures of each mutant are shown as a matrix. Similarly the lowest energy structure is shown. The ThT aggregation assay on WT and mutant tauRD, tau peptides and FL 2N4R was performed as technical triplicates. The data were fit to a non-linear regression model fitting in GraphPad Prism to estimate an average t1/2max with a standard deviation. TEM images were collected twice. The cell experiments were carried out as biological triplicates and the FRET levels were reported as averages with standard deviation.<br><br>Figure 6. N/A. Model derived from the cumulative data in the manuscript.
Randomization	Samples were not allocated into groups, so randomization is not applicable.
Blinding	Samples were not allocated into groups; blinding was not applied.

Reporting for specific materials, systems and methods

We require information from authors about some types of materials, experimental systems and methods used in many studies. Here, indicate whether each material, system or method listed is relevant to your study. If you are not sure if a list item applies to your research, read the appropriate section before selecting a response.

Materials & experimental systems

n/a	Involved in the study
	☒ Antibodies
	☒ Eukaryotic cell lines
	☐ Palaeontology and archaeology
	☐ Animals and other organisms
	☐ Clinical data
	☐ Dual use research of concern

Methods

n/a	Involved in the study
	☐ ChIP-seq
	☒ Flow cytometry
	☐ MRI-based neuroimaging

Antibodies

Antibodies used	Anti-Tau [3-repeat isoform RD3] Antibody, clone 8E6/C11, 05-803, Sigma-Aldrich; Anti-Tau [4-repeat isoform RD4] Antibody, clone 1E1/A6, 05-804, Sigma-Aldrich; Brightvision Poly-HRP-antiMs/Rb/Ra IgG one component (prediluted by manufacturer), DPVO-HRP 55, Immunologic
Validation	The anti-tau antibodies (RD3 and RD4) are recommended for immunohistochemistry applications. The secondary antibody has been validated for detection of HRP in brain tissues.

Eukaryotic cell lines

Policy information about cell lines and Sex and Gender in Research

Cell line source(s)	293T/17 [HEK293T/17] (ATCC CRL-1268), Lenti-X 293T Cell Line (cat # 632180, Takara)
Authentication	Cell lines were not authenticated
Mycoplasma contamination	Cells were confirmed to be free of mycoplasma contamination
Commonly misidentified lines (See ICLAC register)	No misidentified cell lines were used in this study

Flow Cytometry

Plots

Confirm that:

☒ The axis labels state the marker and fluorochrome used (e.g. CD4-FITC).

☒ The axis scales are clearly visible. Include numbers along axes only for bottom left plot of group (a 'group' is an analysis of identical markers).

☒ All plots are contour plots with outliers or pseudocolor plots.

☒ A numerical value for number of cells or percentage (with statistics) is provided.

Methodology

Sample preparation	HEK293 cells stably expressing tauRD fused C-terminally to mEOS3.2 were expressed. A portion of the mEOS was converted with UV, the cells fixed with PFA and analyzed by FACS.
Instrument	BD Fortessa
Software	FlowJo v10
Cell population abundance	at least 10,000 cells were analyzed for each condition in triplicate
Gating strategy	Gates were selected in this order: live cells, FSC singlets, SSC singles, FITC/mCherry positive cells, and FRET positive population

☒ Tick this box to confirm that a figure exemplifying the gating strategy is provided in the Supplementary Information.

This checklist template is licensed under a Creative Commons Attribution 4.0 International License, which permits use, sharing, adaptation, distribution and reproduction in any medium or format, as long as you give appropriate credit to the original author(s) and the source, provide a link to the Creative Commons license, and indicate if changes were made. The images or other third party material in this article are included in the article's Creative Commons license, unless indicated otherwise in a credit line to the material. If material is not included in the article's Creative Commons license and your intended use is not permitted by statutory regulation or exceeds the permitted use, you will need to obtain permission directly from the copyright holder. To view a copy of this license, visit http://creativecommons.org/licenses/by/4.0/
